# Natural Product-Derived Compounds Targeting Keratinocytes and Molecular Pathways in Psoriasis Therapeutics

**DOI:** 10.3390/ijms25116068

**Published:** 2024-05-31

**Authors:** Yu Geon Lee, Younjung Jung, Hyo-Kyoung Choi, Jae-In Lee, Tae-Gyu Lim, Jangho Lee

**Affiliations:** 1 Division of Food Functionality Research, Korea Food Research Institute, Wanju-gun 55365, Republic of Korea; ugun2@kfri.re.kr (Y.G.L.); j.younjung@kfri.re.kr (Y.J.); chkyoung@kfri.re.kr (H.-K.C.); jaeinlee@kfri.re.kr (J.-I.L.); 2 Department of Food Science & Biotechnology, College of Life Sciences, Sejong University, Seoul 05006, Republic of Korea; tglim@sejong.ac.kr; 3 Carbohydrate Bioproduct Research Center, Department of Food Science & Biotechnology, Sejong University, Seoul 05006, Republic of Korea

**Keywords:** psoriasis, keratinocyte, natural products, inflammation, signaling pathway

## Abstract

Psoriasis is a chronic autoimmune inflammatory skin disorder that affects approximately 2–3% of the global population due to significant genetic predisposition. It is characterized by an uncontrolled growth and differentiation of keratinocytes, leading to the formation of scaly erythematous plaques. Psoriasis extends beyond dermatological manifestations to impact joints and nails and is often associated with systemic disorders. Although traditional treatments provide relief, their use is limited by potential side effects and the chronic nature of the disease. This review aims to discuss the therapeutic potential of keratinocyte-targeting natural products in psoriasis and highlight their efficacy and safety in comparison with conventional treatments. This review comprehensively examines psoriasis pathogenesis within keratinocytes and the various related signaling pathways (such as JAK-STAT and NF-κB) and cytokines. It presents molecular targets such as high-mobility group box-1 (HMGB1), dual-specificity phosphatase-1 (DUSP1), and the aryl hydrocarbon receptor (AhR) for treating psoriasis. It evaluates the ability of natural compounds such as luteolin, piperine, and glycyrrhizin to modulate psoriasis-related pathways. Finally, it offers insights into alternative and sustainable treatment options with fewer side effects.

## 1. Introduction

Psoriasis is an autoimmune chronic inflammatory skin disease with a strong genetic component. It affects approximately 2–3% of the global population. It is characterized by persistent inflammation leading to the uncontrolled growth and differentiation of keratinocytes [1]. Psoriasis manifests in several distinct types, each with unique characteristics. Plaque psoriasis (psoriasis vulgaris) is the most common form, characterized by raised, red patches covered with a silvery-white buildup of dead skin cells [2]. Other forms of psoriasis include guttate psoriasis, which is characterized by small, dot-like lesions; inverse psoriasis, which presents with bright red lesions in skin folds; pustular psoriasis, which is marked by white pustules surrounded by red skin; and erythrodermic psoriasis, which leads to widespread redness and severe itching [3,4]. Psoriatic arthritis affects up to 30% of individuals with psoriasis, causing joint pain and stiffness, while nail psoriasis can lead to the pitting and discoloration of the nails [5]. It is worth noting that psoriasis often coexists with psychiatric disorders and psychosocial distress, reducing the quality of life for those affected [6]. Various treatment approaches for psoriasis include the use of topical medications (corticosteroids, retinoids, anthralin, and calcineurin inhibitors), oral drugs, and phototherapy [7]. Topical corticosteroids are the primary choice of intervention, but their prolonged use may lead to skin thinning. For example, anthralin slows skin cell growth but can cause irritation and stains [8]. Retinoids are derived from vitamin A and reduce inflammation, but they have drawbacks such as skin irritation and sun sensitivity. Calcineurin inhibitors are effective but carry a risk of skin cancer and lymphoma. Thus, conventional psoriasis therapies may have disadvantages such as worsening psoriatic symptoms, leading to skin dryness, redness, itching, and damage, especially with prolonged sun exposure [9]. These limitations and side effects warrant the use of natural products to treat psoriasis owing to benefits such as a holistic approach that addresses inflammation with potential gentleness and fewer side effects.

## 2. Pathogenesis of Psoriasis in Keratinocytes

**Figure 1:**
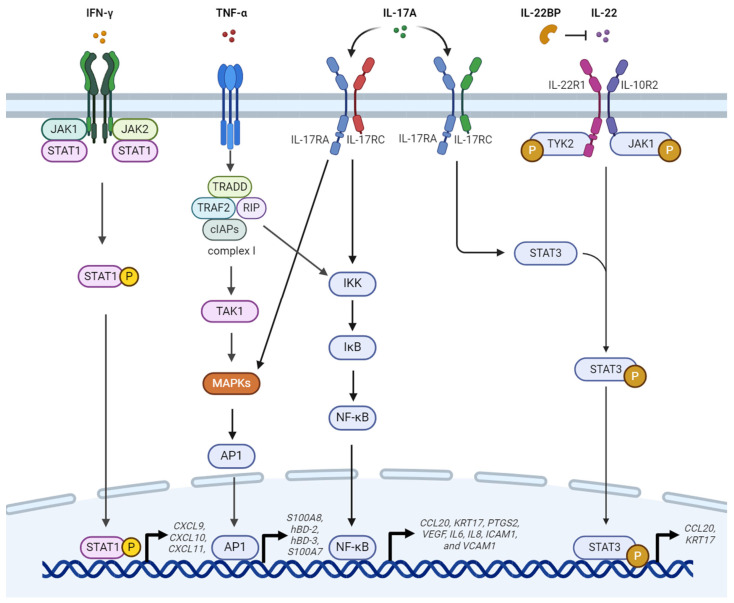
Signaling pathways in psoriasis pathogenesis in keratinocytes. This diagram illustrates the intricate network of cytokine signaling pathways that are activated within keratinocytes by key proinflammatory cytokines such as IL-17A, IL-22, IFN-γ, and TNF-α, which are secreted by various immune cells. The complex interplay of cytokines, signaling cascades, and transcriptional regulators promotes the hyperproliferation, aberrant differentiation, and chronic inflammation observed in psoriatic keratinocytes. IFN-γ binding to its receptor activates the Janus kinase 1/2-signal transducer and activator of transcription 1 (JAK1/2-STAT1) signaling. Phosphorylated STAT1 induces the expression of C-X-C motif chemokine ligand 9 (CXCL9), CXCL10, and CXCL11. Similarly, TNF-α binding to the TNF receptor (TNFR) activates complex I, which comprises TNF receptor-associated death domain (TRADD), TNF receptor-associated factor 2 (TRAF2), receptor-interacting protein, and cellular inhibitor of apoptosis proteins (cIAPs). In turn, these activate the TGF-beta-activated kinase 1 (TAK1), mitogen-activated protein kinases (MAPKs), activator protein 1 (AP1), I-kappa-B kinase (IKK), inhibitor of kappa B (IκB), and nuclear factor-kappa B (NF-κB) pathways. The translocation of AP1 induces S100 calcium-binding protein A8 (S100A8), human beta-defensin 2 (hBD-2), hBD-3, and S100A7 expression, whereas NF-κB activation induces C-C motif chemokine ligand 20 (CCL20), keratin 17 (KRT17), prostaglandin-endoperoxide synthase 2 (PTGS2), vascular endothelial growth factor (VEGF), interleukin 6 (IL-6), IL-8, intercellular adhesion molecule 1 (ICAM1), and vascular cell adhesion molecule 1 (VCAM1) expression. Additionally, IL-17A activates the MAPK, NF-κB, and STAT3 pathways by binding to the IL-17 receptor A/IL-17 receptor C (IL-17RA/IL-17RC) heterodimer. The IL-22 receptor 1/IL-10 receptor 2 (IL-22R1/IL-10R2) heterodimer activates STAT3 by phosphorylating tyrosine kinase 2 (TYK2) and JAK1 and inducing CCL20 and KRT17 expression. Additionally, IL-22 binding protein (IL-22BP) inhibits IL-22 activity by directly binding to it. Image created using BioRender.com (https://www.biorender.com (accessed on 13 March 2024)). Sharp arrows (→) indicate activation, and blunt arrows (⊣) indicate inhibition. Upward arrows (↱) indicate transcriptional activation.

Figure 1 summarizes the signaling pathways that are activated in keratinocytes by cytokines such as interleukin (IL)-17A, IL-22, interferon (IFN)-γ, and tumor necrosis factor (TNF)-α secreted by various immune cells. This section presents an in-depth analysis of the molecular and cellular mechanisms underlying psoriasis pathogenesis, specifically in keratinocytes. Keratinocytes are the predominant cell type of the epidermis; they play a pivotal role in the development and persistence of psoriatic lesions [10,11,12]. This section examines the intricate network of cytokines, signaling cascades, and transcriptional regulators that promote the hyperproliferation, aberrant differentiation, and chronic inflammation observed in psoriatic keratinocytes.

### 2.1. Interleukin-17A (IL-17A)

IL-17A is a prominent member of the IL-17 cytokine family that forms functional homodimers and heterodimers with IL-17F and engages in signaling through the IL-17 receptor family (IL17Rs) [13,14]. IL-17A is a cytokine that is primarily produced by Th17 lymphocytes under the influence of dendritic cell (DC)-derived IL-23. This contributes to psoriasis pathogenesis [15]. The IL-17 receptor (IL-17R) consists of five members. IL-17A-induced activation of IL-17RA and IL-17RC heterodimers leads to the downstream activation of NF-κB, extracellular signal-regulated kinase (ERK), p38 MAPK, and Jun N-terminal kinase (JNK) signaling pathways [16]. In contrast, the activation of IL-17RA and IL-17RD heterodimers by IL-17A primarily activates p38 MAPK and JNK and minimally affects NF-κB and ERK [17]. Additionally, IL-17RA interacts with and transactivates the epidermal growth factor (EGFR), which mediates a complex IL-17A-induced signaling network in psoriasis [18]. Similarly, IL-17A activates the STAT3 pathway in keratinocytes. STAT3 is a crucial signaling molecule in psoriasis development. Transgenic mice expressing constitutively active STAT3 develop a psoriasis-like phenotype, and STAT3 inhibition prevents skin lesion formation [19]. IL-17A triggers STAT3 activation via RIP4, leading to increased CCL20 expression and keratin 17 upregulation. IL-6 and IL-22 work synergistically with IL-17A as potent STAT3 activators in psoriasis [20]. IL-17RA deletion, specifically in keratinocytes, significantly reduces dermatitis, emphasizing its critical role in IL-17A-mediated neutrophil attraction and psoriasis development [21]. IL-36 signaling is crucial in IL-17A-induced psoriasis as it contributes to keratinocyte hyperproliferation and immune cell infiltration. IL-36R inhibition with antibodies effectively suppresses psoriasis-like skin inflammation and attenuates systemic inflammation. This could be a promising alternative treatment for IL-17A-driven psoriasis and associated comorbidities [22]. Moreover, IL-17A induces mitochondrial reactive oxygen species, HIF1α expression, and keratinocyte proliferation. Inhibiting HIF1α or reactive oxygen species reverses these IL-17A-induced metabolic alterations, highlighting the significant role of IL-17A in the metabolic reprogramming of human skin [23]. Beyond psoriasis, the dysregulation of IL-17A has broader implications in various inflammatory and autoimmune diseases. Elevated IL-17A levels are associated with rheumatoid arthritis, multiple sclerosis, and inflammatory bowel disease, highlighting its notable role in immune regulation and inflammation [6]. In rheumatoid arthritis, IL-17A contributes to joint inflammation and damage by stimulating the production of proinflammatory cytokines and matrix metalloproteinases [24]. In multiple sclerosis, IL-17A plays a role in disrupting the blood–brain barrier and recruiting immune cells to the central nervous system, exacerbating neural damage [25]. Patients with inflammatory bowel disease have higher levels of IL-17A, which leads to intestinal inflammation and contributes to tissue damage [26]. Current treatment strategies for managing psoriasis include the use of biologic agents that target IL-17A, such as secukinumab, ixekizumab, and brodalumab. These medications are inhibitors of IL-17A and have been shown to be highly effective in reducing psoriatic lesions and improving patient outcomes. Secukinumab and ixekizumab are monoclonal antibodies that neutralize IL-17A, while brodalumab targets the IL-17 receptor. In addition to improving skin symptoms, these biologics also help reduce systemic inflammation and associated comorbidities in patients with psoriasis [27]. The success of these treatments underscores the therapeutic relevance of IL-17A in psoriasis and its potential as a target in other IL-17A-associated diseases.

### 2.2. Interleukin-22 (IL-22)

IL-22 is a member of the IL-10 family and is significantly elevated in the serum and lesions of patients with psoriasis. IL-22 concentration correlates with disease severity [28]. It is produced by various immune cells, including Th17, Th1, Th22, γδT, and NK cells. IL-22 acts on non-immune cells, particularly keratinocytes, through its receptors IL-22R1 and IL-10R2 [29]. Upon binding, IL-22 activates downstream signals in keratinocytes, inducing antimicrobial protein production and inhibiting keratinocyte differentiation, contributing to psoriasis-like epidermal inflammation [29,30]. IL-22 binding to the epithelial cell-expressed IL-22R activates JAK1 and TYK2, leading to STAT3 phosphorylation and, to a lesser extent, STAT5 phosphorylation. This canonical signaling pathway is integral to IL-22-induced keratinocyte proliferation and migration in psoriasis [31]. Additionally, a non-canonical pathway characterized by phosphotyrosine-independent massive STAT3 activation contributes to IL-22 functions and is implicated in imiquimod (IMQ)-induced psoriasis in mice. Notably, the IL-22-binding protein (IL-22BP), which is a soluble IL-22 receptor, acts as an antagonist by binding to IL-22 with high affinity, which could be a potential regulatory mechanism for IL-22 activity in psoriatic keratinocytes [32]. Dysregulated IL-22 levels in tandem with Th17-cell activation and IL-17 activity are implicated as key factors in psoriasis development. Elevated IL-22 expression in both the skin and peripheral blood of patients with psoriasis compared with that of healthy individuals underscores its involvement in the disease. Specific IL-22 genetic variants are associated with altered skin barrier functions, correlating with an early onset and increased severity of psoriasis. Additionally, impaired IL-22BP production exacerbates skin inflammation in patients with psoriasis [33].

### 2.3. Interferon-γ (IFN-γ)

IFN-γ is a member of a protein family that was initially identified for its non-specific antiviral properties [34]. IFN-γ comprises three classes based on structural and functional criteria. Type I IFNs are induced primarily in response to viral infections and are subdivided into various groups (IFN-α/β/ω) [35]. Type II, which is now known as IFN-γ, is primarily produced by T lymphocytes, NKT cells, and NK cells in response to immune and inflammatory stimuli rather than viral infections [36]. IFN-γ plays a significant role in psoriasis pathogenesis as it is intricately involved in the complex interplay between IL-17 and IFN-γ-producing CD^4+^ and CD^8+^ T-cell subsets [37]. IFN-γ was initially considered a major driver of psoriasis owing to its upregulation in the IL-12/IFN-γ signaling pathway [38]. Elevated IFN-γ levels correlated with disease severity in both serum and skin samples [39]. IFN-γ-induced chemokines, such as CXCL9, CXCL10, and CXCL11, were upregulated in psoriatic lesions [40]. Emerging evidence suggests the involvement of IFN-γ-producing T-cell subsets, particularly in guttate psoriasis, where decreased Tregs may contribute to elevated IFN-γ levels and influence keratinocyte behavior and disease initiation [41]. IFN-γ regulates various cellular functions in psoriatic keratinocytes through the IFN-γ receptor (IFNGR) and the JAK/STAT signaling pathway [42,43]. Upon binding to IFNGR, IFN-γ activates JAK1 and JAK2, leading to the phosphorylation of cytoplasmic STAT1 [44,45]. Phosphorylated STAT1 forms dimers known as IFN-γ-activated factor, which translocate to the nucleus, bind to the IFN-γ activator element, and regulate the transcription of IFN-γ regulatory genes [46]. Additionally, IFN-γ activates the mitogen-activated protein kinase (MAPK) pathway, phosphoinositide 3-kinase pathway, and nuclear factor-kappa B (NF-κB) pathway, contributing to its multifaceted role in psoriasis pathogenesis [47,48,49].

### 2.4. Tumor Necrosis Factor-α (TNF-α)

TNF-α is a key inflammatory cytokine that is highly expressed in psoriatic lesions and plays a pivotal role in psoriasis pathogenesis [50,51]. It is produced by various cells, including keratinocytes, DCs, neutrophils, mast cells, NKT, Th1, Th17, and Th22 cells, and acts through the TNFRI and TNFRII receptors [52]. TNF-α suppresses IFN-α secretion, induces DC maturation, and synergizes with IL-17A to regulate psoriasis-related cytokine and keratinocyte genes [53]. TNF-α facilitates IL-23 release by DCs, promotes T-cell infiltration through interaction with keratinocytes, and collaborates with IL-17 to stimulate inflammatory cytokine and chemokine production in keratinocytes, which amplifies the psoriasis inflammatory cascade [54,55]. In psoriasis, TNF-α primarily engages TNFRI, forming homotrimers that induce intracellular signaling through complex I. This complex involves TNFR1-associated death domain protein (TRADD), TRAF2, receptor-interacting serine/threonine-protein kinase 1 (RIPK1), a cellular inhibitor of apoptosis protein 1 (cIAP1) or cIAP2, and the linear ubiquitin chain assembly complex. The activation of complex I recruits transforming growth factor-β (TGFβ)-activated kinase 1 (TAK1), which activates MAPKs that activate the transcription factor AP1 [55,56]. Additionally, complex I activates IKK via Lys63-linked ubiquitin, leading to the activation of the NF-κB pathway and transcription factor. Both AP1 and NF-κB regulate proinflammatory gene transcription and immune cell proliferation, contributing to psoriasis pathogenesis in keratinocytes [55,57].

## 3. Potential Therapeutic Targets and Signaling Pathways for Psoriasis in Keratinocytes

**Figure 2:**
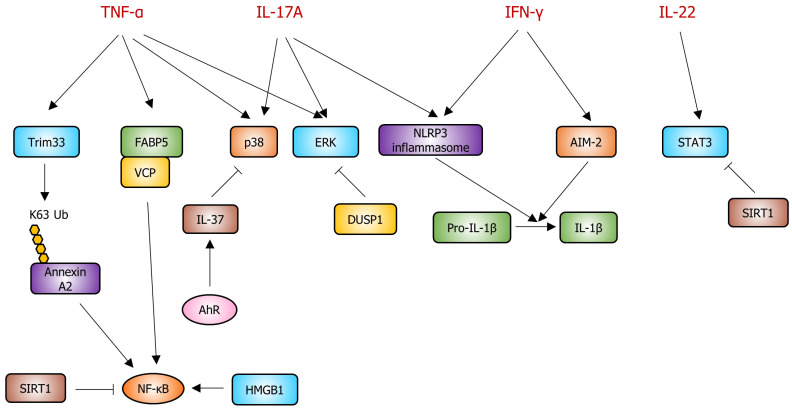
Putative molecular targets and their associated signaling pathways for psoriasis in keratinocytes. TNF-α activates Trim33, which induces K63 ubiquitination of annexin A2. Subsequently, K63 ubiquitination of annexin A2 activates the NF-κB signaling pathway. TNF-α also activates the FABP5-VCP complex, which in turn activates NF-κB. SIRT1 inhibits NF-κB signaling, while HMGB1 activates it. IL-17A or TNF-α activates p38, promoting psoriasis development. AhR increases IL-37 expression, and increased IL-37 inhibits p38 activity. TNF-α or IL-17A activates ERK, while DUSP1 inhibits the ERK pathway. IL-17A or IFN-γ activates the NLRP3 inflammasome, which converts pro-IL-1β to IL-1β. Activated IL-1β is involved in psoriasis pathogenesis. IFN-γ activates AIM-2, which also converts pro-IL-1β to IL-1β. IL-22 activates STAT3, and SIRT1 inhibits STAT3 activity. Sharp arrows (→) indicate activation, and blunt arrows (⊣) indicate inhibition. Yellow circles represent K63 ubiquitination of annexin A2.

In view of the critical involvement of keratinocytes in psoriasis pathology, this section presents promising therapeutic targets within these cells. By dissecting the molecular mechanisms underlying psoriasis in keratinocytes, potential intervention targets can be identified, which would enable the development of innovative treatment strategies. Emphasis has been placed on key signaling pathways such as JAK-STAT, NF-κB, and MAPK and cellular components such as the NLRP3 inflammasome and fatty acid-binding proteins (FABPs). The molecular targets and pathways involved in the pathogenesis of psoriasis are illustrated in Figure 2, and detailed information is presented in Table 1.

### 3.1. The Janus Kinases (JAKs)

JAKs include JAK1, JAK2, JAK3, and TYK2. The JAK pathway is a key cytokine signaling pathway that influences various biological processes [86]. JAK inhibitors such as tofacitinib and ruxolitinib are used to treat autoimmune diseases, including psoriasis [58]. Psoriasis involves the abnormal proliferation and differentiation of keratinocytes and T-cell-mediated inflammatory infiltration. As the JAK-STAT pathway is closely linked to autoimmune diseases, JAK inhibition is currently considered a critical therapeutic target during the development of interventions from natural products. Tofacitinib is a JAK3 inhibitor that has been approved for psoriasis treatment [58]. Ruxolitinib is a JAK1/JAK2 inhibitor that is being evaluated for topical application in psoriasis [59]. The highly selective dual JAK2/FLT3 inhibitor flonoltinib maleate is another potential therapeutic agent [60]. Flonoltinib maleate demonstrated anti-psoriasis activity by inhibiting keratinocyte proliferation, suppressing DC generation and differentiation, and reducing inflammatory factor levels in vitro and in vivo. The compound also reverted pathological changes in psoriasis-like mouse models and reduced inflammatory responses by inhibiting JAK2 and FLT3 signaling pathways [60].

### 3.2. Nuclear Factor-Kappa B (NF-κB)

Nuclear factor-kappa B (NF-κB) is a transcription factor that is a crucial mediator in psoriasis, which is characterized by elevated active phosphorylated NF-κB levels. Genomic studies link psoriasis with NF-κB pathway mediators, suggesting its role in altered keratinocyte and immune cell behavior [87]. Psoriasis pathogenesis involves the canonical NF-κB pathway in response to various stimuli through the polyubiquitination of the IκB kinase (IKK) complex [61]. The non-canonical pathway is activated by specific TNF cytokines and contributes selectively to psoriasis pathogenesis [62]. Susceptibility genes (e.g., *c-Rel*, *TRAF3IP2*, and *CARD14*) and inhibitors (NFKBIA, TNFAIP3, TNIP1, and ZC3H12C) in the NF-κB pathway are associated with psoriasis development [61,88]. Therapies targeting NF-κB, such as TNF-α blockers and glucocorticoids, show promise in psoriasis treatment, but a delicate balance is required to avoid immunodeficiencies [63].

### 3.3. Tripartite Motif-Containing Protein 33 (TRIM33)

TRIM33 is a part of the TRIM E3 ligase family and functions as both an E3 ligase and a transcriptional cofactor. It has various substrates and is involved in tumorigenesis, hematopoiesis, and inflammatory responses. In Th17-cell differentiation, TRIM33 promotes IL-17 expression and IL-10 suppression [64,65]. TRIM33 is upregulated in psoriatic epidermis. Its overexpression in keratinocytes promotes the expression of psoriasis-linked proinflammatory cytokines. TRIM33 induces the K63-linked ubiquitination of annexin A2 (ANXA2), resulting in the interaction of TRIM33 with the p50/p65 NF-κB subunits. Thus, p50/p65 is retained in the nucleus, promoting the expression of inflammation-related NF-κB downstream genes. The study suggests TRIM33 as a potential target for psoriasis treatment due to its pivotal role in keratinocyte inflammation through the ANXA2/NF-κB pathway [66].

### 3.4. NLRP3 Inflammasome

The NLRP3 inflammasome is a component of the inflammasome complex. It responds to stimuli such as pathogens and proinflammatory cytokines. Among various inflammasomes, the NLRP3 inflammasome has been extensively studied and is associated with inflammatory diseases. The NLRP3 inflammasome plays a crucial role in psoriasis, particularly in keratinocytes [89]. The peripheral blood levels of inflammasome-related proteins are elevated in patients with psoriasis [67]. Genetic variations in inflammasome-related genes are associated with psoriasis susceptibility [68]. Psoriasis involves immune cell-induced skin inflammation with an overexpression of proinflammatory cytokines, notably those secreted by Th17 cells. In psoriasis, pathogenic T cells activate the NLRP3 inflammasome in keratinocytes, leading to the cleavage of pro-IL-1β by caspase-1 and the production of active IL-1β, which exacerbates skin inflammation [67]. In psoriatic keratinocytes, AIM2 and NLRP3 are upregulated by IFN-γ, while DNA sensing via AIM2 triggers IL-1β release [68]. Levels of negative regulators such as CARD18 are increased in non-lesional skin [90]. Thus, therapeutic agents that target the inflammasome hold promise for the treatment of psoriasis.

### 3.5. Fatty Acid-Binding Protein (FABP)–Valosin-Containing Protein (VCP) Complex

FABPs are a family of small proteins that facilitate fatty acid transport and cellular responses. This family comprises at least nine members that play pivotal roles in obesity-associated diseases. FABP5 has been identified in epidermal tissues, regulates keratinocyte function, and is implicated in skin inflammation and chronic lesions. Recent studies suggest a potential little-known role for FABP5 in psoriasis pathogenesis, which contributes to the understanding of its involvement in various diseases [69]. The FABP–VCP complex is implicated in psoriasis for promoting NF-κB signaling and neutrophil infiltration. Elevated FABP5 levels in psoriatic skin contribute to the recruitment of neutrophils, which is a hallmark of psoriasis. FABP5 interacts with VCP, which is a crucial player in NF-κB activation, and their silencing inhibits NF-κB signaling and neutrophil chemotaxis, suggesting a role in the molecular mechanisms underlying psoriasis pathogenesis [70].

### 3.6. High-Mobility Group Box-1 (HMGB1)

High-mobility group box 1 (HMGB1) is a nuclear protein that is released by activated macrophages. HMGB1 contributes to inflammation by inducing proinflammatory responses through Toll-like receptor 4 (TLR4). Additionally, the release of HMGB1 through autophagy in keratinocytes promotes skin inflammation in psoriasis [91,92]. The keratinocytes in the skin lesions of psoriasis model mice showed increased HMGB1 expression. Keratinocyte-derived HMGB1 promotes inflammatory macrophage activation, which increases the proportion of M1-type macrophages in skin lesions. However, the local clearance of macrophages alleviates psoriasis-like inflammation. Additionally, HMGB1 promotes excessive keratinocyte proliferation and inflammatory cytokine expression [71]. The proinflammatory effect of HMGB1 was studied by treating normal human keratinocytes with recombinant HMGB1, and 11 inflammatory factors were found to be upregulated, with IL-18 exhibiting the most significant change. The activation of NF-κB signaling and inflammasomes mediates HMGB1-induced IL-18 expression [72]. This highlights HMGB1 as a potential target for psoriasis treatment in keratinocytes.

### 3.7. Sirtuins (SIRTs)

Sirtuins (SIRTs) are evolutionarily conserved enzymes involved in diverse post-translational modifications, including deacetylation and polyADP-ribosylation. SIRTs are encoded by the yeast *Sir2* gene and play crucial roles in DNA recombination, gene silencing, and various biological processes such as transcriptional regulation and oxidative stress modulation. Human SIRT1, which is the closest homolog of yeast Sir2, has seven homologs with distinct cellular localizations and exerts functions in the nucleus, cytoplasm, and mitochondria [73]. SIRTs are implicated in psoriasis pathogenesis. Psoriasis shows reduced SIRT1-5 expression and increased SIRT6 and SIRT7 expression [74]. Activating SIRT1 mitigates oxidative stress-induced damage, which inhibits MAPK, NF-κB, and STAT3 pathways [73]. Modulating SIRT activity using SIRT1-5 agonists and SIRT6-SIRT7 inhibitors is a potential therapeutic option for psoriasis treatment.

### 3.8. Aryl Hydrocarbon Receptor (AhR)

AhR, also known as the dioxin receptor, binds to environmental polyaromatic hydrocarbons and dioxins, triggering oxidative stress through reactive oxygen species (ROS) generation. AhR is a versatile receptor that is activated by various exogenous and endogenous ligands, including photo-induced chromophores, phytochemicals, and microbial bioproducts [93]. AhR is implicated in psoriasis, particularly in regulating the immune balance of Th17/22 and Treg cells. In an IMQ-induced psoriasis model, AhR deficiency played a key role in exacerbating skin inflammation (including in keratinocytes) with nonhematopoietic cells [75,93]. The activation of AhR with FICZ and tapinarof has demonstrated therapeutic effects by modulating the expression of cytokines, specifically IL-17a, IL-17f, IL-19, IL-22, IL-23a, and IL-1b [75,76,77]. AhR also regulates the expression of IL-33 and IL-37, which influences the balance between inflammatory and anti-inflammatory responses. AhR agonists such as tapinarof and galactomyces ferment filtrate (GFF) activate the AhR/IL-37 axis in keratinocytes and swiftly downregulate IL-33 by inhibiting p38 MAPK phosphorylation. This is a potential therapeutic approach for psoriasis. [78,79]. In May 2022, tapinarof was approved by the FDA for psoriasis treatment as a topical cream following successful clinical trials that confirmed its effectiveness [94,95]. These effects are AhR-dependent, and highlight the potential of AhR modulation as a therapeutic strategy for psoriasis.

### 3.9. Mitogen-Activated Protein Kinases (MAPKs)

MAPKs include p38 MAPK, ERK, and c-Jun NH2-terminal kinase (JNK). They form signaling pathways that are crucial in psoriasis pathogenesis. The activation of ERK1/2, p38, and JNK MAPKs has been observed in psoriatic lesions, indicating their involvement in psoriasis pathogenesis [55,80]. In psoriasis, the JNK pathway is activated in keratinocytes by various signals. This pathway regulates inflammatory cytokine and chemokine production, mediates immune cell recruitment, and influences keratinocyte proliferation and differentiation. Additionally, JNK is implicated in Th1/Th17-cell recruitment, cytokine production, and FOXP3 regulation for Treg development [81]. The p38 MAPK pathway is crucial as it regulates the production of S100A8, hBD-2, hBD-3, S100A7, and CCN1-induced IL-1β in keratinocytes [82,83]. Moreover, ERK signaling through MSK1 and ERK1/2 phosphorylation contributes to proinflammatory cytokine expression [84]. The negative regulator DUSP1/MKP-1 is downregulated in psoriasis, and its overexpression inhibits keratinocyte proliferation by targeting the ERK pathway [85]. CARD14 mutations in psoriasis induce inflammatory cytokines through the MALT1-mediated aberrant activation of NF-κB and JNK signaling pathways [82].

### 3.10. Dual-Specificity Phosphatase-1 (DUSP1)

Dual-specificity phosphatase-1 (DUSP1) is a member of the dual-specificity phosphatase family that is involved in inactivating MAPK isoforms. It plays varied roles in cell processes and exhibits differential effects in different cancers [96]. In psoriasis, DUSP1 expression is notably reduced in psoriasis-inducing cocktail-treated dermal mesenchymal stem cells and HaCaT cells. DUSP1 overexpression suppresses HaCaT cell proliferation by downregulating cyclin D1 and Rb and enhances apoptosis, as evidenced by increased cell apoptosis rate, c-caspase 3 levels, and the BAX/BCL-2 ratio. Mechanistically, DUSP1 exerts its effects through the ERK/ELK-1/EGR-1 signaling pathway, as enhanced DUSP1 expression reduces p-ERK, p-ELK-1, and EGR-1 protein levels. Chromatin immunoprecipitation assays reveal p-ELK-1 binding to the EGR-1 promoter, and EGR-1 overexpression abolishes the regulatory roles of DUSP1 [85]. This study highlights the involvement of DUSP1 in regulating proliferation and apoptosis in HaCaT cells and offers insights into its potential role in psoriasis pathogenesis.

## 4. Natural Product-Derived Compounds for Psoriasis Therapeutics

**Figure 3:**
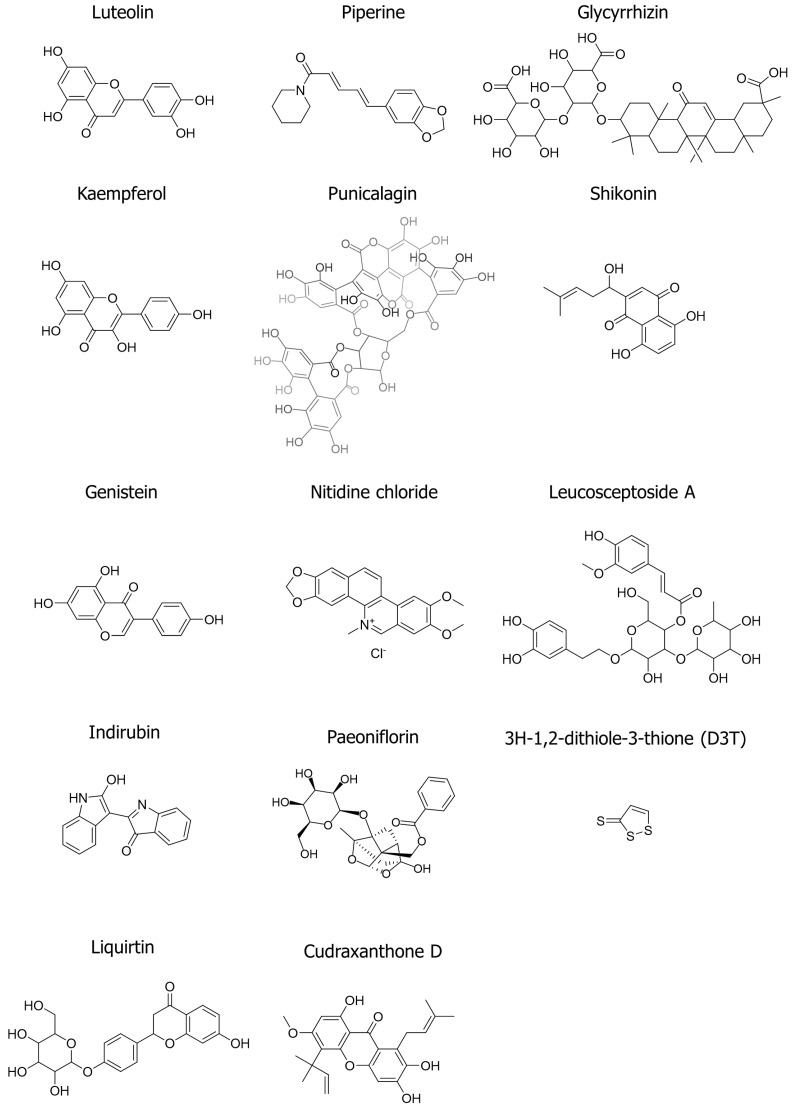
Chemical structures of natural product-derived compounds used in psoriasis treatment. The figure shows the chemical structures of 14 different compounds (luteolin, piperine, glycyrrhizin, kaempferol, punicalagin, shikonin, genistein, nitidine chloride, leucosceptoside A, indirubin, paeoniflorin, 3H-1,2-dithiole-3-thione (D3T), liquirtin, and cudraxanthone D). The chemical structures were generated using ChemDraw (Ver. 23.1.1).

**Figure 4:**
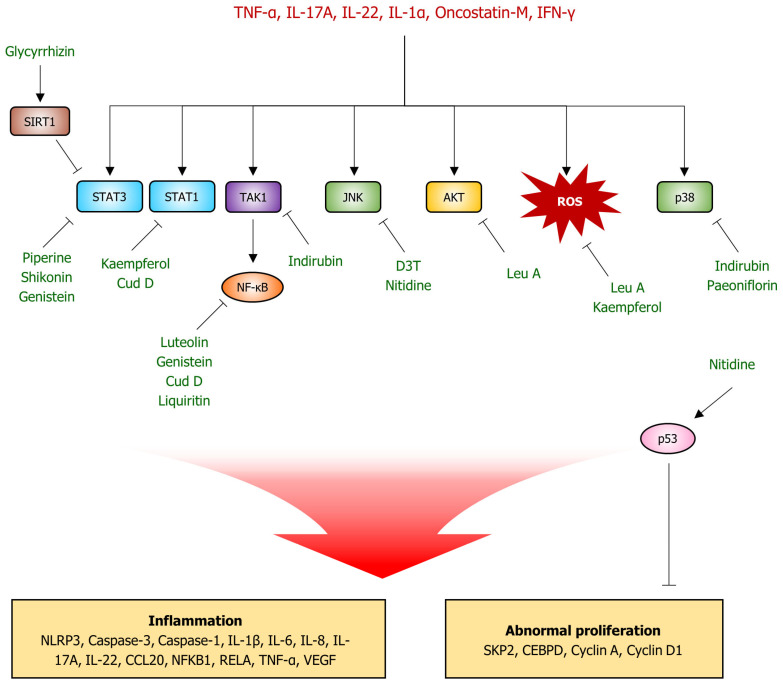
Potential molecular targets of natural product-derived compounds for psoriasis treatment. TNF-α, IL-17A, IL-22, IL-1α, Oncostatin-M, and IFN-γ promote the activation of STAT3, STAT1, TAK1/NF-kB, JNK, AKT, and p38 signaling systems, as well as ROS production. These signaling pathways and ROS production lead to inflammatory responses by increasing the expression or protein levels of NLRP3, caspase-3, caspase-1, and others in keratinocytes. They also contribute to abnormal keratinocyte proliferation by increasing the levels of SKP2, CEBPD, cyclin A, and cyclin D1. Piperine, shikonin, and genistein inhibit STAT3, while glycyrrhizin inhibits STAT3 activity through SIRT1 activation. Kaempferol and cudraxanthone D (Cud D) also inhibit STAT1 activity. Luteolin, genistein, Cud D, and liquirtin inhibit NF-kB transactivity, whereas indirubin inhibits NF-kB signaling by inhibiting TAK1 activity. 3H-1,2-dithiole-3-thione (D3T) and nitidine inhibit the JNK signaling pathway, a member of the MAPK signaling system, and indirubin and paeoniflorin inhibit p38, another signaling pathway. Leucosceptoside A (Leu A) inhibits the PI3K/AKT signaling pathway, while Leu A and kaempferol inhibit ROS overproduction. Furthermore, p53 is known to inhibit excessive keratinocyte proliferation, and nitidine activates the p53 pathway. Sharp arrow (→) indicates activation, and blunt arrow (⊣) indicates inhibition.

The previous sections established the complex signaling networks that contribute to psoriasis. This section presents natural products with the potential to modulate these pathways. Natural compounds possess diverse pharmacological properties; hence, they can potentially target key inflammatory mediators, signaling cascades, and cellular processes implicated in psoriasis. This section explores 14 natural products (luteolin, piperine, glycyrrhizin, kaempferol, punicalagin, shikonin, genistein, nitidine chloride, leucosceptoside A, indirubin, paeoniflorin, 3H-1,2-dithiole-3-thione, liquirtin, and cudraxanthone D), their underlying mechanisms of action within keratinocytes, and their potential to alleviate the symptoms of psoriatic lesions. The chemical structures of these compounds are shown in Figure 3. Additionally, Table 2 presents a summary of the therapeutic efficacy of natural compounds in the treatment of psoriasis, as evidenced by in vitro and in vivo studies. Figure 4 illustrates the putative molecular targets of each compound and the signaling pathways involved in the treatment of psoriasis using these compounds.

### 4.1. Luteolin

Luteolin is a flavone present in vegetables, fruits, and medicinal herbs. It is synthesized through the phenylpropanoid pathway in plants. It is known for its health-promoting effects, such as antioxidant, anti-inflammatory, hepatoprotective, anticancer, and neuroprotective properties [114]. Luteolin plays a crucial role in addressing psoriasis. Luteolin has demonstrated potent anti-inflammatory properties in keratinocytes by inhibiting the TNF-α-induced production of inflammatory mediators such as IL-6, IL-8, and VEGF. Additionally, it reduces the TNF-α-induced phosphorylation, nuclear translocation, and DNA binding of NF-κB, which is a key factor in inflammation-mediated transcription. Moreover, luteolin decreases the mRNA expression of NF-κB subunits and effectively curtails keratinocyte proliferation, which is a distinctive feature of psoriatic skin [97].

### 4.2. Piperine

*Piper longum* is a medicinal herb used in Traditional Chinese Medicine (TCM) for various therapeutic purposes, including analgesia and treating stomach disease, coronary heart disease, and stroke. Piperine is the most abundant alkaloid derived from *P. longum* and belongs to the amide class of alkaloids. It imparts the characteristic hot and pungent flavor to *P. longum* and has been officially recognized as a labeling compound for the quality control of *P. longum*. Piperine shows significant pharmacological potential to treat psoriasis [98] by exhibiting therapeutic effects such as ameliorating psoriatic skin lesions, reducing epidermal hyperplasia, inhibiting inflammatory cell infiltration, and decreasing the expression of characteristic psoriasis-associated cytokines and chemokines. Its inhibition of STAT3 phosphorylation further contributes to its anti-inflammatory effects in psoriatic skin inflammation and offers new strategies for psoriasis treatment [99].

### 4.3. Glycyrrhizin

Glycyrrhizin is also known as glycyrrhizic acid. It is a triterpenoid saponin found in licorice (*Glycyrrhiza glabra*) and imparts the sweet taste to licorice. Glycyrrhizin exists in various forms, such as potassium and calcium salts of 18β-glycyrrhetinic acid. Its chemical structure contributes to the diverse chemical composition of licorice and is the principal component responsible for its therapeutic properties [115]. Moreover, glycyrrhizin exhibits notable pharmacological potential, including anti-inflammatory, hepatoprotective, anti-carcinogenic, and anti-viral properties. These properties are attributed to its active metabolite, glycyrrhetinic acid. Glycyrrhetinic acid has shown anti-inflammatory effects by targeting TLR-4 and has demonstrated the ability to inhibit TMPRSS2, a protein involved in viral entry [116]. Glycyrrhizin has demonstrated therapeutic potential for psoriasis by alleviating adverse symptoms in an IMQ-induced mouse model, improving the pathological state of skin cells, and inhibiting IL-17A and IFN-γ expression. In vitro studies using IL-17A-treated HaCaT cells showed that glycyrrhizin suppressed cell proliferation and reversed the IL-17A-induced expression of inflammatory markers (IL-6, CCL20, and TNF-α) through SIRT1 upregulation and STAT3 signaling inhibition. These findings suggest that glycyrrhizin may mitigate psoriasis by modulating immune responses and keratinocyte proliferation via the IL-17A/SIRT1/STAT3 axis [100].

### 4.4. Kaempferol

Kaempferol is a flavonoid named after Engelbert Kaempfer. It is a polyphenolic compound that is found in various plants, particularly in *Camellia sinensis* (tea tree). Kaempferol is a member of the flavonol subclass of the flavonoid group, which is the largest group of secondary plant metabolites. It plays essential roles in plant growth, regulation, and defense. It is known for its antioxidant properties and is associated with a range of health benefits, including hepatoprotective, antimicrobial, renoprotective, anti-diabetic, cardioprotective, anti-arthritic, neuroprotective, gastroprotective, and anti-mutagenic effects [117]. Kaempferol exhibits therapeutic potential for psoriasis by modulating immune-mediated inflammation. In HaCaT cells, it reduces intracellular ROS production, inhibits rhIFN-γ-induced IFN-γR1 expression, upregulates SOCS1 levels, and inhibits the phosphorylation of JAK-STAT signaling molecules. In an IMQ-induced psoriasis-like mouse model, it alleviated skin lesions, reduced DC numbers and γδT17 cell population, and downregulated proinflammatory cytokine levels. This highlights its multifaceted effects on the JAK-STAT pathway and immune response in psoriasis [101].

### 4.5. Punicalagin

Punicalagin is a polyphenol that is predominantly found in *Punica granatum* (pomegranate), particularly in the form of 2,3-hexahydroxydiphenoyl-gallagyl-D-glucose. It is recognized for its high antioxidant content. Recent studies have indicated its potential neuroprotective effects, as evidenced by its protection against vincristine-induced neuropathic pain in rats. It has been studied as an herbal drug with neuroprotective properties, encouraging further research into its therapeutic and preventive applications for neurodegenerative diseases [118]. Punicalagin directly inhibited abnormal keratinocyte proliferation induced by inflammatory factors such as TNF-α, IL-17A, and IL-6 by suppressing S-phase kinase-associated protein 2 (SKP2) expression in both in vitro HaCaT cells and in vivo models, suggesting its potential as a promising treatment for psoriasis [102].

### 4.6. Shikonin

Shikonin is a natural naphthoquinone compound found in the roots of the *Boraginaceae* family. It is known for its antioxidant, anti-tumor, antifungal, and anti-inflammatory properties and has been used in TCM. Shikonin and its derivatives have potential applications in food, cosmetics, and pharmacology. Shikonin is being investigated for treating immune-related diseases like psoriasis and rheumatoid arthritis owing to its diverse pharmacological effects, including anti-inflammatory actions [119,120] and immunosuppressive properties. It has demonstrated potential in treating psoriasis by inhibiting the JAK/STAT3 pathway, which counteracts IL-17-induced effects and suppresses VEGF expression. In HaCaT cells and an IMQ-induced psoriasis mouse model, shikonin restored tumor suppressor CCAAT/enhancer-binding protein δ (CEBPD) expression [103]. It inhibited IL-17-induced VEGF expression in keratinocytes and suppressed the JAK2/STAT3 pathway. These results suggest its therapeutic potential in addressing psoriasis pathogenesis by targeting CEBPD and IL-17 and exerting anti-angiogenic effects in psoriatic lesions [104].

### 4.7. Genistein

Genistein is a primary isoflavone in soybeans and comprises > 60% of soy isoflavones. It functions as a phytoestrogen with potential therapeutic applications. It exhibits a range of pharmacological effects, including antioxidant, anti-inflammatory, anti-apoptotic, and anti-angiogenic properties. Several studies have highlighted its impact on various diseases and emphasized its potential role in treating conditions such as postmenopausal symptoms, cancer, and cardiovascular diseases [121,122]. Genistein has demonstrated anti-psoriatic effects by reducing epidermal thickness and inhibiting inflammatory factors in an IMQ-induced psoriasis-like mouse model. In vitro, it hampered keratinocyte proliferation and suppressed TNF-α-induced inflammation through STAT3 inhibition and NF-κB signaling modulation. Thus, genistein shows promise for treating psoriasis lesions by targeting inflammation [105].

### 4.8. Nitidine Chloride

Nitidine chloride is a quaternary ammonium benzophenanthridine alkaloid that was first isolated in 1959 from *Zanthoxylum nitidum* roots. It has subsequently been discovered in various medicinal plants but is predominantly extracted from *Z. nitidum* in TCM. It is known for multiple traditional therapeutic effects and pharmacological activities, including anti-tumor, anti-inflammation, anti-colitis, anti-malaria, and anti-osteoporosis effects [123,124]. Nitidine chloride has emerged as a potent inhibitor of human HaCaT keratinocytes, exhibiting effectiveness in inhibiting cell proliferation and inducing S-phase cell cycle arrest. The underlying inhibitory mechanisms involve reduced DNA synthesis, an altered expression of cell cycle-associated proteins, and apoptosis induction through the c-JNK pathway. Notably, in both TPA- and IMQ-induced epidermal hyperplasia and inflammation models, nitidine chloride demonstrated efficacy in reducing tissue thickness, weight, and inflammation, making it a promising candidate for psoriasis treatment with potential anti-inflammatory and antiproliferative effects [106].

### 4.9. Leucosceptoside A

Leucosceptoside A is a phenylethanoid glycoside that belongs to a group of natural compounds synthesized through the shikimic acid and cinnamate pathways. It is derived from the base compound verbascoside and is distinguished by a methyl group in its caffeic acid moiety. It exhibits unique structural features and notable biological activities [125], including therapeutic potential against psoriasis. In an in vitro model using IFN-γ/IL-17A/IL-22-stimulated HaCaT cells, leucosceptoside A effectively mitigated psoriasis-related inflammation by suppressing the PI3K/AKT signaling pathway. This highlights its potential as a regulator of keratinocyte differentiation in the treatment of psoriasis [107].

### 4.10. Indirubin

Indirubin is a bisindole alkaloid with the molecular formula (3-(3-oxo-1H-indol-2-ylidene)-1H-indol-2-one). It occurs widely in various sources and is also found in Indigo naturalis and traditional Chinese medicines. Indirubin and its derivatives are known for their superior anti-tumor and anti-inflammatory effects with reduced toxicity; hence, they are of significant interest in leukemia therapy [126]. Indirubin demonstrates anti-inflammatory effects by strongly inhibiting chemokine CCL20 expression and secretion in IL-17A-stimulated keratinocytes. This inhibition is mediated primarily through the TAK1 signaling pathway, as observed in both a mouse psoriasis-like model and cultured HaCaT cells. By disrupting the CCL20/CCR6 axis-mediated inflammatory loops, indirubin effectively ameliorates psoriasiform dermatitis. This study highlights the potential of indirubin as a therapeutic agent for psoriasis and provides valuable mechanistic insights [108].

### 4.11. Paeoniflorin

Paeoniflorin is a valuable natural product that is primarily extracted from the roots of *Paeoniaceae* plants. It is the main constituent of the total glucosides of paeony and is of significant medicinal importance because it exhibits therapeutic potential against cardiovascular and neurological conditions. Recent studies have highlighted its anti-inflammatory effects in targeting TLR-mediated signaling and its promising outlook in treating pain, cerebral ischemic injury, neurodegenerative diseases, heart attack, diabetic kidney issues, and atherosclerosis [127]. It exhibits therapeutic potential for treating psoriasis by ameliorating lesions and decreasing the Baker Score in a psoriasis-like guinea pig model. In vitro, paeoniflorin significantly inhibited IL-6, IL-17A, and IL-22 mRNA expression with a marginal effect on IL-17A and IL-6 protein expression but notable inhibition of IL-22 protein expression. This inhibitory effect is attributed to the suppression of p38 MAPK phosphorylation. In a mouse model, paeoniflorin reduced macrophage and neutrophil infiltration, which suppressed cytokine production and inflammatory marker levels [109]. This immunomodulatory effect of paeoniflorin provides insights into its therapeutic potential for alleviating psoriatic skin lesions, suggesting that paeoniflorin may act as an active component of the total glucosides of paeony with therapeutic implications for psoriasis [110].

### 4.12. 3H-1,2-dithiole-3-thione (D3T)

3H-1,2-dithiole-3-thione (D3T) is derived from cruciferous vegetables. In the context of psoriasis, D3T exhibits significant anti-inflammatory effects by reducing skin inflammation and modulating Th17 cell differentiation. In an IMQ-induced psoriasis mouse model, D3T treatment notably reduced ear thickness, skin redness, scaling, and the expression of KI-67, the NLRP3 inflammasome, and cleaved caspase-1 in skin samples. Furthermore, D3T inhibited Th17 differentiation, decreased IL-6 and IL-17A levels in serum samples, and suppressed the expression of NLRP3, caspase-1, and IL-1β in TNF-α-stimulated HaCaT cells. The mechanistic study reveals that D3T exerted these effects by inhibiting the JNK pathway and highlights the potential of D3T as a promising agent for psoriasis treatment by targeting NLRP3 inflammasome activation [111].

### 4.13. Liquiritin

Liquiritin is a flavonoid from Radix et Rhizoma Glycyrrhizae. It is used in TCM and has been reviewed for its pharmacokinetics and diverse pharmacological effects, including anti-Alzheimer’s, antidepressant, anti-tumor, anti-inflammatory, cardiovascular protection, antitussive, hepatoprotection, and skin protective properties [128]. Liquiritin demonstrates significant potential as a therapeutic agent for psoriasis in both in vitro (using TNF-α-stimulated HaCaT keratinocytes) and in vivo (IMQ-induced psoriasis-like mouse model) settings, where it effectively inhibits psoriasis progression. It suppressed HaCaT keratinocyte proliferation without affecting cell viability, alleviated skin inflammation, and reduced Th17 and DC accumulation. In mice, it diminished NF-κB and AP-1 signal pathways [112]. Mechanistically, liquiritin downregulates inflammatory cytokine expression in keratinocytes. These findings collectively suggest that liquiritin may be a pivotal regulator in treating psoriasis by modulating key signaling pathways.

### 4.14. Cudraxanthone D

Cudraxanthone D (CD) is derived from the root of *Cudrania tricuspidata*. It is a natural xanthone known for its anti-inflammatory, neuroprotective, and antioxidant effects [129]. In an IMQ-induced mouse model and TNF-α/IFN-γ-activated keratinocytes, CD significantly reduced key psoriatic indicators such as skin thickness, Psoriasis Area Severity Index score, and neutrophil infiltration. Moreover, CD suppressed TNF-α, immunoglobulin G2a, and myeloperoxidase serum levels and inhibited Th1/Th17-cell expression in splenocytes. In TNF-α/IFN-γ-activated keratinocytes, CD suppressed inflammatory cytokine expression by modulating STAT1 phosphorylation and NF-kB nuclear translocation [113]. These findings highlight the potential of CD as a therapeutic candidate for managing psoriasis.

## 5. Conclusions, Perspectives, and Limitations

Psoriasis is a complex chronic inflammatory disease characterized by the intertwining of genetic susceptibility, immune system dysregulation, and environmental triggers. The pathological cascade within keratinocytes involves a complex interplay of cytokines, aberrant signaling pathways, and dysregulated cellular processes. Although conventional therapies provide a degree of relief, their limitations underscore the need for innovative and complementary approaches. Natural products and their diverse bioactive compounds offer a rich source of potential modulators with the capacity to target multiple aspects of psoriasis pathogenesis. This review has presented natural compounds with the potential to suppress proinflammatory signaling pathways, including JAK/STAT, NF-κB, and the NLRP3 inflammasome, and modulate keratinocyte proliferation and differentiation. Compounds such as luteolin and piperine have demonstrated compelling activity in preclinical models, which evidences their potential to reduce inflammation, scaling, and the overall severity of psoriatic lesions. Despite the promising outlook of the research on natural products for treating psoriasis, several challenges remain to be addressed. Nonetheless, the evidence presented in this review underscores the untapped potential of natural products as a source of novel, sustainable, and potentially safer therapeutic agents for the management of psoriasis. Natural product-derived compounds used for psoriasis therapeutics encounter various limitations. These compounds often lack specificity and end up affecting multiple signaling pathways instead of directly targeting the specific pathways responsible for keratinocyte hyperproliferation in psoriasis. Furthermore, their low bioavailability, resulting from poor solubility, permeability, and stability, hampers their efficacy when administered orally or topically. Additionally, while certain natural compounds have shown anti-psoriatic effects in vitro or in animal models, their potency may not be sufficient to produce clinically relevant effects in humans, particularly for moderate to severe cases. Moreover, natural compounds can interact with conventional psoriasis treatments and other medications, which can result in adverse effects or reduced efficacy. The lack of comprehensive clinical data, along with a shortage of large-scale, well-controlled trials, further limits their adoption in clinical practice. To address these limitations, researchers are currently working on developing novel drug delivery systems, combination therapies, and standardized formulations. These advancements aim to improve the bioavailability, specificity, and potency of natural compounds for psoriasis treatment. It is crucial to conduct more rigorous clinical studies in order to establish the therapeutic potential of these promising natural product-derived compounds.

## Figures and Tables

**Table 1:** Molecular targets and pathways in psoriasis: An overview of key signaling mechanisms and therapeutic agents.

Target	Expression or Activity	Signaling Pathways	Inhibitors or Agonists	References
JAKs (JAK1, JAK2, JAK3, TYK2)	- IL-22 activates JAK1 and Tyk2 through phosphorylation - IFN-γ activates JAK1 and JAK2 through phosphorylation	- IL-22 increases the phosphorylation of JAK1 and Tyk2, resulting in an increase in STAT3 phosphorylation - IFN-γ leads to an increase in JAK1 and JAK2 phosphorylation, resulting in an increase in STAT1 phosphorylation	- Inhibitors: Tofacitinib, a JAK3 inhibitor - The dual JAK2/FLT3 inhibitor, flonoltinib maleate	[58,59,60]
NF-κB	- Canonical: activated by various cytokines, such as IL-17A - Non-canonical: primarily activated by TNF cytokines	- IL-17A triggers IKK phosphorylation, leading to IκB phosphorylation and NF-κB activation - TNF-α triggers TRADD/RIP/TRAF2 complex I activation, leading to IKK/IκB phosphorylation and NF-κB activation	Inhibitors: tumor necrosis factor-α blockers; glucocorticoids; BAY 11-7082	[61,62,63]
TRIM33	TRIM33 expression increases in the epidermis of patients with psoriasis	- K63-linked ubiquitination of annexin A2 (ANXA2) increases, leading to NF-κB pathway activation - IL-17 expression increases and IL-10 expression decreases, leading to Th17 cell activation	-	[64,65,66]
NLRP3 inflammasome	- Blood levels of inflammasome-related proteins increase in patients with psoriasis - Activated by Th17 cytokines and IFN-γ	- Th17 cytokines activate NLRP3 inflammasome, leading to an increase in pro-IL-1β cleavage and IL-1β release - IFN-γ activates AIM2, leading to an increase in IL-1β release	Inhibitor: BAY11-7082 (inhibiting the NLRP3 inflammasome activity and the NF-κB pathway)	[67,68]
FABP-VCP complex	FABP5 expression increases in the epidermal tissues of patients with psoriasis	FABP5 expression increases, interacting with VCP, ultimately leading to NF-κB pathway activation	-	[69,70]
HMGB1	HMGB1 expression increases in keratinocytes of a psoriasis mouse model	HMGB1 expression increases, leading to NF-κB pathway activation and an increase in IL-18 expression	-	[71,72]
SIRTs	- SIRT1-5 expression decreases in psoriasis - SIRT6 expression increases in psoriasis	SIRT1 activation decreases the activity of the MAPK, NF-κB, and STAT3 pathways	SIRT1 activator: resveratrol; catapol	[73,74]
AhR	Activated by AhR ligands	IL-37 expression increases, decreasing p38 pathway activity and IL-33 expression	Agonists: Tapinarof, galactomyces ferment filtrate (GFF)	[75,76,77,78,79]
MAPKs	MAPKs (ERK, p38, and JNK) are activated in psoriatic lesions	- JNK activation increases proinflammatory cytokine expression and Th1/Th17 cell recruitment - p38 activation increases the production of S100A8, hBD-2, hBD-3, S100A7, and IL-1β; MSK1/ERK activation increases proinflammatory cytokine expression	- JNK inhibitor: SP600125 - p38 inhibitor: SB203580 - ERK inhibitor: U0126	[55,80,81,82,83,84]
DUSP1	DUSP1 expression decreases in keratinocytes	ERK/ELK-1/EGR-1 pathway activity increases	-	[85]

**Table 2:** Therapeutic efficacy of natural compounds on psoriasis: in vitro and in vivo studies.

Compound	Experimental Models	Inducers for Psoriasis	Concentrations of the Compounds	Effects of the Compounds	Reference
Luteolin	HaCaT and primary keratinocyte	In vitro: 50 ng/mL TNF-α	10–100 μM in vitro	Luteolin demonstrated a reduction in proliferation and a decrease in the expressions of IL-6, IL-8, VEGF, NFKB1, and RELA mRNA levels in vitro.	[97]
Piperine	HaCaT in vitro; BALB/c mice (male, 8–10 weeks old) in vivo	In vitro: M5 (10 ng/mL) containing TNF-α, IL-17A, IL-22, IL-1α, and Oncostatin-M; in vivo: 62.5 mg of 5% IMQ cream	10–40 μM in vitro; 2 mM and 4 mM in vivo (topical)	Piperine exhibited a reduction in S100A7 protein levels and mRNA expression of IL-6, IL-23, β-defensin 2, and CCL20 both in vitro and in vivo. Moreover, it decreased the levels of various cytokines and mRNAs associated with psoriasis in vivo.	[98,99]
Glycyrrhizin	HaCaT in vitro; BALB/c mice (male, 10 weeks old) in vivo	In vitro: 100 ng/mL IL-17A; in vivo: 62.5 mg of 5% IMQ cream	2 μM in vitro; 20 mg/kg in vivo (topical)	Glycyrrhizin decreased the proliferation and secretion of IL-6, CCL20, and TNF-α in vitro, while increasing the expression of SIRT1 and reducing STAT3 phosphorylation. Additionally, it reduced the secretion of IL-17A and IFN-γ in the serum in vivo.	[100]
Kaempferol	HaCaT in vitro; BALB/c mice (female, 10 weeks old) in vivo	In vitro: IFN-γ (500 U/mL); in vivo: 62.5 mg of 5% IMQ cream	5–40 μM in vitro; 9.01, 27.03, and 81.09 mg/kg in vivo (oral)	Kaempferol decreased JAK-STAT phosphorylation, ROS production, and IFN-γR1 expression while increasing SOCS1 expression in vitro. In vivo, it reduced the number of dendritic cells in the skin.	[101]
Punicalagin	HaCaT in vitro	IL-6 (50 ng/mL), IL-17A (12.5 ng/mL), and TNF-α (12.5 ng/mL)	2.5–160 μM in vitro	Punicalagin reduced abnormal proliferation, SKP2 expression, and the cytokine-enhanced S-phase fraction in vitro.	[102]
Shikonin	HaCaT in vitro; BALB/c (male, 8 weeks old) in vivo	In vitro: IL-17A (40 ng/mL); in vivo: 50 mg of 5% IMQ cream	5 μM in vitro; 1 μM in vivo (topical)	Shikonin reduced VEGF expression, JAK/STAT3 pathway activity, CEBPD expression, and keratinocyte abnormal proliferation both in vitro and in vivo.	[103,104]
Genistein	HaCaT in vitro; BALB/c (male, 7–8 weeks old)	In vitro: 20 ng/mL TNF-α;in vivo: 62.5 mg of 5% IMQ cream	0.5% and 2% in vivo (topical)	Genistein decreased TNF-α-induced proliferation, inflammatory factor expression, STAT3 phosphorylation, and NF-κB signaling in vitro. It also reduced epidermal thickness and the expression of inflammatory factors in vivo.	[105]
Nitidine chloride	HaCaT in vitro; BALB/c mice (female, 6–8 weeks old) in vivo	In vivo: 20 μL TPA (50 μg/mL per site); 62.5 mg of 5% IMQ cream	7.8 nM in vitro; 1.5 μg (topical) in vivo	Nitidine chloride inhibited S-phase cell cycle arrest caused by cell proliferation, decreased the expression of cyclin A and cyclin D1, increased p53 expression and apoptosis, and enhanced JNK phosphorylation both in vitro and in vivo.	[106]
Leucosceptoside A	HaCaT in vitro	IFN-γ, IL-17A, and IL-22 (1 ng/mL each)	20 μM in vitro	Leucosceptoside A inhibited PI3K/AKT signaling and reduced the expression of STAT3, PI3KCA, and AKT mRNAs in vitro.	[107]
Indirubin	HaCaT in vitro; BALB/c mice (male, 8 weeks old) in vivo	In vitro: 100 ng/mL IL-17A;in vivo: 42 mg of 5% IMQ cream	4–16 μM in vitro; 12.5, 25, and 50 mg/kg in vivo (topical)	Indirubin decreased CCL20 expression; TAK1-mediated NF-κB signaling; and p38 and MKK4 phosphorylation both in vitro and in vivo. Moreover, it reduced ki67-positive cells in vivo.	[108]
Paeoniflorin	HaCaT in vitro; BALB/c mice (female, 8–11 weeks old) or Hartley guinea pigs (male, 4 weeks old) in vivo	In vitro: 5 μg/mL LPS; in vivo: 0.2 mL of 5% propranolol cream (guinea pigs) or 60 mg of 5% IMQ cream (BALB/c mice)	6.24–104.07 μM in vitro; 9% paeoniflorin emulsion (topical, guinea pigs), 75, 150, or 300 mg/kg/day (topical, mice)	Paeoniflorin reduced IL-6, IL-17A, and IL-22 secretion and mRNA expression in vitro. It also decreased p38 phosphorylation, propranolol chloride-induced parakeratosis, and hyperkeratinization in vivo. Moreover, it alleviated IMQ-induced psoriatic symptoms, inflammation, and cytokine production in vivo.	[109,110]
3H-1,2-dithiole-3-thione (D3T)	HaCaT in vitro; BALB/c (female, 8 weeks old) in vivo	In vitro: 10 ng/mL TNF-α; in vivo: 62.5 mg of 5% IMQ cream	50–200 μM in vitro; 10 and 30 mg/kg in vivo (intraperitoneal)	D3T reduced the expression of NLRP3, caspase-1, and IL-1β in vitro and attenuated JNK pathway activity. In vivo, it decreased ear thickness; skin redness; scaling; ki-67 levels; and NLRP3 and inflammasome levels, while cleaving caspase-1, IL-6, and IL-17A. Moreover, it inhibited Th17 differentiation.	[111]
Liquirtin	HaCaT in vitro; C57BL/6 (female, 8–10 weeks) in vivo	In vitro: 10 ng/mL TNF-α; in vivo: 62.5 mg of 5% IMQ cream	5, 10, and 20 μM in vitro; 1 and 2 mg/kg in vivo (intragastric)	Liquirtin reduced TNF-α-induced proliferation and the mRNA expression of IL-6, IL-8, and IL-1β, as well as the activation of NF-κB and AP-1 pathways in vitro. In vivo, it diminished psoriasis-like phenotypes and the expression of IL-6, TNF-α, IL-23, and IL-17A, as well as the population and polarization of Th17 cells.	[112]
Cudraxanthone D	HaCaT in vitro; C57BL/6 (female, 8 weeks old) in vivo	In vitro: 10 ng/mL TNF-α + 10 ng/mL IFN-γ; in vivo: 62.5 mg of 5% IMQ cream	0.01–1 μM in vitro; 0.1, 1, and 10 mg/kg in vivo (intragastric)	Cudraxanthone D reduced the expression of CCL17, IL-6, IL-8, and IL-1β in vitro and inhibited the NF-κB and STAT1 pathways. In vivo, it alleviated inflammation in psoriasis-like skin and reduced the expression of CXCL1, IL-6, and IL-4.	[113]

## Data Availability

No new data were created or generated in this manuscript. Data sharing is not applicable to this article.

## Abbreviations

AhR: aryl hydrocarbon receptor, AIM2: Absent in Melanoma 2, ANXA2: Annexin A2, AP1: Activator Protein 1, CARD14: Caspase Recruitment Domain Family Member 14, CCL20: C-C Motif Chemokine Ligand 20, cIAP: Cellular Inhibitor of Apoptosis Protein, CXCL: C-X-C Motif Chemokine Ligand, CXCL9, CXCL10, CXCL11: C-X-C Motif Chemokine Ligands 9, 10, 11, DC: dendritic cell, D3T: 3H-1,2-dithiole-3-thione, IMQ: imiquimod, DUSP1: Dual-Specificity Phosphatase-1, EGFR: Epidermal Growth Factor Receptor, FABP: Fatty Acid-Binding Protein, FICZ: 6-Formylindolo(3,2-b)Carbazole, GFF: Galactomyces Ferment Filtrate, HMGB1: High-Mobility Group Box-1, ICAM1: Intercellular Adhesion Molecule 1, IFNGR: Interferon-γ Receptor, IFN-γ: Interferon-γ, IKK: I-kappa-B Kinase, IL-1β: Interleukin-1β, IL-6: Interleukin-6, IL-8: Interleukin-8, IL-17A: Interleukin-17A, IL-22: Interleukin-22, JAK: Janus Kinase, MAPK: Mitogen-Activated Protein Kinase, MALT1: Mucosa-Associated Lymphoid Tissue Lymphoma Translocation Protein 1, NLRP3: NOD-Like Receptor Family Pyrin Domain Containing 3, NK: natural killer, NF-κB: Nuclear Factor-kappa B, PTGS2: Prostaglandin-Endoperoxide Synthase 2, RIPK1: Receptor-Interacting Serine/Threonine-Protein Kinase 1, ROS: Reactive Oxygen Species, SIRT: Sirtuin, SKP2: S-phase kinase-associated protein 2, STAT: Signal Transducer and Activator of Transcription, STAT3: Signal Transducer and Activator of Transcription 3, Th17: T Helper 17, TAK1: TGF-beta-Activated Kinase 1, TCM: Traditional Chinese Medicine, TLR: Toll-like receptor, TLR4: Toll-like receptor 4, TNF-α: Tumor Necrosis Factor-α, TRADD: TNF Receptor-Associated Death Domain, TRAF2: TNF Receptor-Associated Factor 2, TRIM33: Tripartite Motif-Containing Protein 33, Tregs: Regulatory T Cells, VCAM1: Vascular Cell Adhesion Molecule 1, VCP: Valosin-Containing Protein, VEGF: Vascular Endothelial Growth Factor.
